# Conducting a psychosocial and lifestyle assessment as part of an integrated care approach for childhood obesity: experiences, needs and wishes of Dutch healthcare professionals

**DOI:** 10.1186/s12913-021-06635-6

**Published:** 2021-06-28

**Authors:** L. W. Koetsier, M. M. A. van Mil, M. M. A. Eilander, E. van den Eynde, C. A. Baan, J. C. Seidell, J. Halberstadt

**Affiliations:** 1 Department of Health Sciences, Faculty of Science, Vrije Universiteit Amsterdam, Amsterdam Public Health Research Institute, Amsterdam, The Netherlands; 2 Erasmus MC, University Medical Center Rotterdam, Obesity Center CGG, Rotterdam, The Netherlands; 3 Tilburg University, Tranzo, Tilburg School of Social and Behavioural Sciences, Tilburg, The Netherlands

**Keywords:** Qualitative research, Childhood obesity assessment, Integrated care approach, The Netherlands, Healthcare professionals

## Abstract

**Background:**

The causes and consequences of childhood obesity are complex and multifaceted. Therefore, an integrated care approach is required to address weight-related issues and improve children’s health, societal participation and quality of life. Conducting a psychosocial and lifestyle assessment is an essential part of an integrated care approach. The aim of this study was to explore the experiences, needs and wishes of healthcare professionals with respect to carrying out a psychosocial and lifestyle assessment of childhood obesity.

**Methods:**

Fourteen semi-structured interviews were conducted with Dutch healthcare professionals, who are responsible for coordinating the support and care for children with obesity (coordinating professionals, ‘CPs’). The following topics were addressed in our interviews with these professionals: CPs’ experiences of both using childhood obesity assessment tools and their content, and CPs’ needs and wishes related to content, circumstances and required competences. The interviews comprised open-ended questions and were recorded and transcribed verbatim. The data was analysed using template analyses and complemented with open coding in MAXQDA.

**Results:**

Most CPs experienced both developing a trusting relationship with the children and their parents, as well as establishing the right tone when engaging in weight-related conversations as important. CPs indicated that visual materials were helpful in such conversations. All CPs used a supporting assessment tool to conduct the psychosocial and lifestyle assessment but they also indicated that a more optimal tool was desirable. They recognized the need for specific attributes that helped them to carry out these assessments, namely: sufficient knowledge about the complexity of obesity; having an affinity with obesity-related issues; their experience as a CP; using conversational techniques, such as solution-focused counselling and motivational interviewing; peer-to-peer coaching; and finally, maintaining an open-minded, non-stigmatizing stance and harmonizing their attitude with that of the child and their parents.

**Conclusions:**

Alongside the need for a suitable tool for conducting a psychosocial and lifestyle assessment, CPs expressed the need for requisite knowledge, skills and attitudes. Further developing a supporting assessment tool is necessary in order to facilitate CPs and thereby improve the support and care for children with obesity and their families.

**Supplementary Information:**

The online version contains supplementary material available at 10.1186/s12913-021-06635-6.

## Background

### Childhood obesity

Childhood obesity is a growing public health concern that affects children in both developed and developing countries [1]. In 2019, 3.3% of children aged 2–9 years and 2.3% of children aged 9–18 in the Netherlands had obesity [2, 3]. Childhood obesity challenges both the physical and psychosocial health of the child. For example, it is associated with an increased prevalence of cardiometabolic risk factors, metabolic complications and physical impairments, as well as with low self-esteem, anxiety and an increased risk of depression [4–10]. Research demonstrates that children with obesity are likely to suffer from obesity later in life, which, in turn, increases the risk of other chronic diseases [11, 12]. Hence, adequate support and care is required for children with obesity, in order to help both them and their families make the necessary sustainable lifestyle changes to improve their health, societal participation and quality of life [13, 14].

### Obesity guidelines

Given that the causes and consequences of childhood obesity are complex and multifaceted, it is necessary to utilize a tailored approach that recognizes this complexity [15]. Both national and international guidelines have been developed for managing obesity amongst children and adolescents [14, 16–20]. These obesity guidelines seek to establish what constitutes adequate support and care for children with obesity, as well as specify what the requirements are for both the diagnosis and management of childhood obesity. Although these guidelines on childhood obesity are commonly respected, implementation is often insufficient [21]. For instance, Dutch healthcare professionals encounter a wide range of barriers in their daily healthcare practices, such as insufficient early identification of overweight, high drop-out rates amongst children who are receiving treatment, allied with difficulties in helping families to bring about long-term behaviour change [13, 22].

The support and care, as well as the indicated prevention that is outlined in the Dutch obesity guidelines takes place across two domains. The first is the social domain, where youth healthcare (YHC) professionals play a central role in monitoring all children’s health, and, as such, are also involved in the early identification of overweight and obesity. Their services form part of the YHC system that is provided free of charge to all families and falls under the responsibility of municipal health services. This system also includes social care, such as support from social workers, child psychologists and community workers who attempt to encourage exercise and sports in schools and neighbourhoods. The second domain is healthcare, in which professionals such as general practitioners, paediatricians and dietitians provide the care. For children, this care is covered under the national basic health insurance [23, 24].

### Integrated care for childhood obesity

The Dutch government has designated measures in the national prevention agreement to reduce the prevalence of childhood overweight and obesity and improve the care for children with obesity as one of its key ambitions [25]. This ambition also extends to targeting children who are at a high risk of obesity due to having overweight. The ‘National model for integrated care for childhood overweight and obesity’, which was co-developed by eight selected Dutch municipalities, was published in 2018 [13]. This model, which is based on both scientific and practice-based evidence, delineates a processual approach to the provision of integrated care for children with overweight and obesity (aged 0–19 years). The national model functions as a guidebook for implementing integrated care within local municipalities and comprises a six-step process: (1) ‘diagnose obesity’, (2) ‘conduct a broad assessment’, (3) ‘discuss the interrelatedness of factors and determine which approach to take’, (4) ‘design a care plan that includes agreements with the child, the parents and other professionals ’, (5) ‘begin to execute the care plan’, and (6) ‘make sure the resulting changes are sustained’.

According to the national model, coordinating professionals (CPs) have an important role to play. The CPs are responsible for connecting the multidisciplinary network of local professionals, for coordinating and monitoring the care provision and progress of the child and their family, for motivating and supporting them, and initiating all of the necessary steps. The role of the CP can be fulfilled by a range of professionals working in different disciplines [13, 26].

### Assessing childhood obesity

There are two elements involved in conducting a broad assessment (step 2 in the process): (1) a physical examination coordinated by the CP, including the actual identification of obesity and an assessment of the weight-related physical health risks; (2) an assessment of a family’s psychosocial circumstances and lifestyle factors carried out by an CP [13, 18, 27]. In this paper, psychosocial problems encompass a combination of psychological factors (such as self-image, mood or well-being) and social factors (such as contact with peers, school or authorities) and the interaction between them [28]. Although both these elements tend to intertwine in practice, this study specifically focuses on the assessment of psychosocial and lifestyle factors, which provides insight into (a) the factors that play a role in the development and maintenance of obesity (e.g. (the psychological) wellbeing of the child and the parents and the family-dynamics), (b) the extent to which the child and the parents are able to manage the challenges and the degree of support that they need, and (c) the lifestyle behaviours of both the child and family (nutrition, exercise and sleep).

### Aim of the study

An assessment of psychosocial and lifestyle factors forms part of a continuum within a processual integrated care approach and is necessary for offering a tailored approach that corresponds to the needs, goals and opportunities of the child and the family. Moreover, it is an important step in terms of both improving support and care and bolstering compliance, which, in turn, will produce better long-term sustainable outcomes for children and their families. In addition, there is a need for knowledge from health care professionals in order to conduct this specific step. No previous studies have investigated what factors facilitate CPs in conducting a psychosocial and lifestyle assessment. Consequently, this study aims (1) to explore the experiences of CPs with regard to conducting an assessment of psychosocial and lifestyle factors as part of an integrated care approach for childhood obesity, and (2) to identify the needs and wishes of CPs with respect to conducting this assessment.

## Methods

### Study design

This study utilized a qualitative approach, which involved conducting semi-structured face-to face interviews with CPs employed in Dutch municipalities that were in different phases of implementing the Dutch ‘National model for integrated care for childhood overweight and obesity’ [13]. The COREQ (Consolidated Criteria for Reporting Qualitative research) was used to describe the methods [29].

### Participants

25 CPs from nine municipalities, along with two project leaders from two municipalities that were already partly in the network of the research team, were approached face-to-face, by telephone or by email to take part in the study. A key criterion for participation was prior experience in conducting a psychosocial and lifestyle assessment

Ultimately, fourteen CPs from nine municipalities participated in the study. One CP cancelled the interview due to personal circumstances. Other reasons for non-participation were: ‘limited time’, ‘insufficient experience of conducting a psychosocial and lifestyle assessment’, and ‘a colleague from the same municipality is already participating’. It was planned to interview 16 CPs, after 13 interviews the interviewers did not retrieve new information based on their impressions, which was confirmed by the 14th interview. Therefore, data saturation was achieved. An overview of the characteristics of the interviewees is presented in Table 1*. General characteristics of the CPs.*

**Table 1:** General characteristics of the CPs

N	Age (years)	Function	Gender	Organization	Experience as a CP
CP^a^ 1	30	YHC nurse^b^ for children aged 0–12 years	Female	Municipal health service	Two years
CP 2	63	YHC nurse for children aged 0–19 years	Male	Municipal health service	Three years
CP 3	37	YHC nurse for children aged 0–19 years	Female	Municipal health service	Four years
CP 4	56	YHC nurse for children aged 0–19 years	Female	Municipal health service	Few weeks
CP 5	54	YHC nurse for children aged 0–19 years	Female	Municipal health service	Few weeks
CP 6	26	YHC nurse for children aged 0–19 years	Female	Municipal health service	Five months
CP 7	49	YHC nurse for children aged 4–19 years	Female	Municipal health service	One and a half years
CP 8	54	YHC nurse for children aged 4–19 years	Female	Municipal health service	Three months
CP 9	39	YHC nurse for children aged 4–12 years	Female	Municipal health service	Three/four years
CP 10	64	YHC nurse for children aged 0–18 years	Female	Municipal health service	Eight years
CP 11	45	Child health coach	Female	Self-employed coach	Five years
CP 12	56	YHC nurse for children aged 12 years and older	Female	Municipal health service	Three years
CP 13	46	YHC nurse for children aged 0–12 years	Female	Municipal health service	Two months
CP 14	58	YHC nurse for children aged 0–18 years	Female	Municipal health service	Three months

^a^*CP* coordinating professional

^b^*YHC nurse* youth health care nurse

### Setting and data collection

The semi-structured interviews took place between November 2019 and February 2020 at a location chosen by the CP (most commonly in their consultation room). On average, the interviews lasted one hour and were conducted by two female researchers (LK, MvM) with prior training in carrying out qualitative research. A semi-structured interview guide was developed and pilot-tested by LK, MvM and ME, in order to: (1) explore the experiences of CPs with regard to conducting a psychosocial and lifestyle assessment as part of an integrated care approach for childhood obesity, and (2) identify the needs and wishes of CPs with respect to conducting a psychosocial and lifestyle assessment as part of an integrated care approach (*see* Table 2*. Interview topic guides for CPs and the themes that emerged out of the interviews)*. LK wrote field notes both during and after the interviews. All interviews were audio recorded and transcribed verbatim. For the purposes of member checking, summaries of the interviews were provided to participants for them to make comments and corrections if needed.

**Table 2:** Interview topic guides for CPs and the themes that emerged out of the interviews

Research question	
1. Experiences of CPs with regard to conducting a psychosocial and lifestyle assessment as part of an integrated care approach for childhood obesity	**Interview topic guide** (a) CPs’ attitudes towards their role and the vision of the national model (b) Caseload of the CP and perceived differences in the provision of support and care for children with respectively overweight and obesity (c) Conducting a psychosocial and lifestyle assessment; methods used, by whom, how, and where? (d) Themes to discuss with the child and parent when carrying out a psychosocial and lifestyle assessment (e) The (coordinating) process after carrying out a psychosocial and lifestyle assessment (f) Facilitators and barriers related to conducting a psychosocial and lifestyle assessment
	**Identified themes by extracting key words and codes from the coding framework** a) Initiating a psychosocial and lifestyle assessment b) Assessment of psychosocial and lifestyle factors c) CPs’ attitudes towards their role
2. Wishes and needs of CPs with respect to conducting a psychosocial and lifestyle assessment as part of an integrated care approach for childhood obesity	**Interview topic guide** (a) Needs related to the content of psychosocial and lifestyle assessments (b) Needs related to the amount of time and the preferred location for conducting a psychosocial and lifestyle assessment (c) Competences needed to conduct a psychosocial and lifestyle assessment, including the perceived ability needed to conduct a psychosocial and lifestyle assessment
	**Identified themes by extracting key words and codes from the coding framework** a) Support in initiating and conducting a psychosocial and lifestyle assessment b) Competences needed by the CP in order to conduct a psychosocial and lifestyle assessment

### Data analysis

A template analysis was used to analyse the interviews [30]. Template analysis is a form of thematic analysis, in which the development of a coding framework is central, but yet the style and format of the framework is flexible. In template analysis, a set of a priori themes from a sub-set of data or existing theory provides input for the initial framework, which is complemented with open coding. The coding framework is then revised and refined when applied to further data. Two researchers (LK, MvM) coded independently, before checking the consistency of the coding and discussing any discrepancies until a consensus was reached. The preliminary set of codes was discussed by the entire research team and subsequently modified. The key words and codes of the coding framework were assigned by two researchers independently, in order to identify themes and extract content from the data (LK, MvM). The data were analysed using MAXQDA (version 2020).

## Results

The reporting of the results is structured according to the two main research questions, under which in total five themes emerged out of the analysis of the interviews (*see* Table 2*. Interview topic guides for CPs and the themes that emerged out of the interviews).*

### (1) The experiences of coordinating professionals with regard to conducting a psychosocial and lifestyle assessment

The findings that emerged out of the analysis of the interviews regarding the first research question were divided into the three most prominent themes: (a) initiating a psychosocial and lifestyle assessment, (b) assessment of psychosocial and lifestyle factors, and (c) CPs’ attitudes towards their role.

#### (a) Initiating a psychosocial and lifestyle assessment

In order to establish the appropriate tone and focus when discussing a child’s weight status, CPs indicated that it was important to get to know the family first, such as, for example, by exploring the attitude of both the child and their primary care givers (in most cases their parents) towards the child’s weight, or, alternatively, by anticipating the reactions of the child and the parents. The CPs also stressed the need to be delicate with the words one chooses, in order to avoid a disconnection with the child and family, showing a lack of disrespect or stigmatizing the child and family. Given that CPs considered the child’s weight to be a sensitive topic, they were often concerned that discussing it with the children and their parents could induce conflict or alienate them from the family. Therefore, they felt that the conversation should not primarily focus on the child’s weight, especially in the case of children with overweight. With regard to children with obesity, seven CPs expressed that it was more urgent to discuss the weight status of the child; indeed, two CPs even mentioned that they found conversations about weight easier with children with obesity because of the attendant health risks. Five other CPs drew no distinction between overweight and obesity when it came to initiating a conversation about weight. *“I do find obesity concerning because it’s a health risk, too, right?... So, I do talk to the parents about it in the presence of the child. They should be aware of the health risks, if they are not already, as well as the consequences if they don’t take any action.” (CZV 13).*

Four CPs used cards with predefined topics to help initiate the conversation. To increase the engagement of the child and parents during the conversation, they used a range of materials including videos about healthy lifestyles and posters about maintaining a healthy weight, eating healthy food and portion control. Finally, CPs emphasized the need to spend enough time with children and their parents, in order to establish rapport and build a trusting relationship. Indeed, they felt that trust is absolutely paramount if children and their parents are to talk about sensitive topics without feeling ashamed or judged, and that it reduces the risk of drop-out. *“So, invest in mutual trust. That’s very important to me. And that usually works quite well... as long as you build rapport slowly at first, you will be able to achieve more and more.” (CZV5 5).*

#### (b) Assessment of psychosocial and lifestyle factors

If the child and their parents agree to attend a follow-up meeting regarding the child’s weight, CPs plan a separate consultation to conduct an assessment of the psychosocial and lifestyle factors. In particular, CPs expressed that the following topics were important to discuss with the child and their family: the child’s wellbeing, school-related factors, bullying, social environment (e.g. friends), home environment and lifestyle (nutrition, physical exercise and sleep behaviour). Depending on the child’s age (predominantly with children aged 12 years and older), themes such as self-image and potential eating disorders were also considered to be essential to discuss. Additional questions for the parents focused on their financial situation and whether they were experiencing any parenting issues.

All fourteen CPs used a pre-existing supporting assessment tool, which had been developed locally by two municipalities. This tool encompasses three themes: (1) child factors (physical factors, family history, psychological factors and social participation), (2) family factors (system dynamics and parental related problems), and (3) lifestyle factors (nutrition, physical exercise, sleep behaviour and enjoyment) [31].

Given the comprehensiveness of these themes, most CPs believed that this assessment tool provided a complete and broad picture of both the child and their family’s living circumstances. More specifically, they noted that the supporting assessment tool enabled them to gain important insight into the potential underlying causes of the child’s obesity, which, in turn, helped them provide customized support and care to children and their families based on their needs, goals and opportunities**.** *“Well, I use the supporting assessment tool to ask for just about anything I can think of outside of the kilos. Anything from how their health is, how’s school going, do they enjoy school, what kind of sports do they do, do they enjoy going there? Just ask as many questions as needed to get an idea of the life of the child in question. But, mainly, what does the whole family situation look like? (CZV 2)”.*

Twelve CPs argued that a home visit was the most suitable setting to conduct the assessment of psychosocial and lifestyle factors, as the family’s home environment shed light on the family dynamics and the family’s living circumstances. Upon either families’ request or for practical reasons, this assessment typically took place at the consultation office. Most of the CPs needed one hour to assess the psychosocial and lifestyle factors, while three CPs required one and a half hours. In most cases, both the child and their parent(s) attended the consultation, albeit the active participation of the child was dependent on their age and ability to engage in conversation. CPs reported that other people, such as family members or acquaintances, were also able to attend this conversation if desired.

#### (c) CPs’ attitudes towards their role

The majority of CPs were positive about their role as CPs. They especially appreciated the broad perspective needed for this role, whereby the context of the child and family have to be taken into account, as well as the long-term involvement that they have with children and their families. *“Often there will be a whole backstory there. Whether it’s an entire system of poverty or parents who are separated; I think those are important factors to take into account.” (CZV 4).*

Some CPs indicated that they already fulfilled the CP role in their capacity as YHC nurses and thus knew the families for a long time, which, in turn, enhanced the quality of the contact with the child and their family. Conversely, two CPs perceived the CP role to be a challenging one, while two others mentioned that the role is not yet fully clarified and needs to be specified. Five CPs expressed having a lack of time to fulfil their role properly, particularly as it often took considerable time to build rapport and a trusting relationship with children and their parents. Three of them (all within the same municipality) had to fulfil the CP role alongside their YHC nurse tasks, which added to their workload. *“Getting things off to a good start can be challenging, and even after that it can continue to be tricky to get everyone on board and working towards the same goals. But I really enjoy it. Particularly because the interaction with the parents and children is not the same as it is in a consultation room.” (CZV 9)*

### (2) Needs and wishes of coordinating professionals with respect to conducting a psychosocial and lifestyle assessment

The findings emerging out of our analysis of the interviews regarding the second research question were divided into the two most prominent themes: (a) support to initiate and conduct a psychosocial and lifestyle assessment, and (b) competences required by the CP to conduct a psychosocial and lifestyle assessment.

#### (a) Support to initiate and conduct a psychosocial and lifestyle assessment

##### (a.1) materials to initiate a psychosocial and lifestyle assessment

CPs commented that certain materials were critical for facilitating the conversation about psychosocial and lifestyle factors. CPs had varying opinions about which materials were most helpful. Seven CPs reported a need for more appropriate visual materials, which, in conjunction with other materials, can assist children and their families to both formulate their thoughts and increase their awareness of what is deemed to be a healthy weight and a healthy lifestyle. Moreover, CPs felt these materials could enhance both children’s and parents’ knowledge and understanding of health and well-being, alongside educating them about the child’s weight status. Furthermore, because visual materials are generally appealing to children, these materials can also help to facilitate a child-friendly atmosphere, which was seen as an important prerequisite for having an open and honest conversation. CPs reported that younger children in particular benefitted from playful and visually-oriented conversations (for example, via playing board games or memory-based games like Matching Pairs together about obesity-related issues), although these activities should be tailored to the age of the child. Other CPs stressed a need for materials that guide the conversation through a series of pre-determined topics, in order to facilitate dialogue on obesity, healthy living or specific problems that go beyond food and lifestyle. Lastly, to facilitate the conversation with families who either have low literacy or language barrier, CPs felt that using icons that clarify the meaning of the topics being discussed would be helpful. *“When there’s a considerable language barrier and I wonder whether they fully understand me, I sometimes use pictures and symbols for support, because they can be very useful.” (Transcript CZV 13, Pos. 110).*

##### (a.2) materials to structure a psychosocial and lifestyle assessment

When discussing the content of the developed supporting assessment tool, CPs indicated that an improved version would be desirable. Specifically, CPs mentioned the following improvements: adding in-depth questions for specific themes (such as parenting issues); adding themes suitable for children of high school age (such as peer pressure and body-image); optimizing the sequence of the themes; and explaining the objectives of the questions. Alongside this, CPs expressed the need for an accompanying instructional guide explaining how to use the supporting assessment tool. Three CPs also conveyed that a lack of time and language-related barriers negatively impacted on their ability to use the supporting assessment tool, as these issues were not conducive to an in-depth conversation. *“Yes, that can sometimes be very challenging. Sometimes it’s a bit like a game of questions and answers. And sometimes a real conversation happens. But that depends on the parent too.... and, of course, language also plays a role.” (CZV 9).*

The CPs also indicated that it would be helpful if the reporting of the findings in the child’s medical record were simplified, and that a clear and structured working method was currently lacking in this regard. The findings of the psychosocial and lifestyle assessment were also reported differently in children’s medical records, and, hence, CPs stated that they would benefit from integrating the supporting assessment tool within the children’s medical records. *“It’s very useful that the system offers a consultation specifically for this purpose, and that you can click on a case history and that a case history header automatically pops up there with the questions that you would normally have them answer electronically, or on paper, or electronically in another document. So, everything is right there where you want it.” (CZV 6)*

#### (b) Competences required by CPs to conduct a psychosocial and lifestyle assessment

Remarks pertaining to the competences required by CPs to conduct a psychosocial and lifestyle assessment were organized into the following subthemes: knowledge, skills and CPs’ attitudes.

The CPs expressed that having knowledge about the causes and consequences of overweight and obesity was of vital importance, both for being able to initiate the conversation with confidence and to properly explain the complexity of obesity to the child and parents. Most CPs considered overweight and obesity as complex, having extensive experience as CP and displaying an affinity with the issues related to overweight and obesity was deemed to be helpful. *“See, you need to have a feel for that. If it’s not your thing, you shouldn’t do it. Because people will pick up on it.” (CZV 9).*

CPs spoke about how conversational techniques, such as solution-focused counselling and motivational interviewing, also helped to facilitate the process of conducting an assessment. Although CPs learned about these techniques during their nursing education and CP training, their working practices would benefit from continuing education in these techniques, as it would help them to communicate confidently with children and parents. CPs felt they also would benefit from additional training and peer-to-peer coaching on specific cases. *“Peer review is something I personally find very important. I think it’s great to hear from each other how you tackle things and decide for yourself, ‘oh yes that’s a good fit in this case or not at all.’” (CZV 13).*

CPs regularly perceived resistance from both the child and their parents when addressing their weight. In light of this, the CPs stressed how important it was to both strike the right tone in the conversation and to build rapport and a trusting relationship. To achieve the latter, CPs noted that it was vital to adopt an open and non-stigmatizing stance and to harmonize their attitude with that of the child and their parents. Moreover, engaging in active listening, using probing open questions and displaying a non-patronizing attitude were also considered to be expedient. *“I think it’s how you approach people first and foremost. Every parent does their best to raise their children well and so.... start from that perspective and share positive observations. And avoid sitting opposite a client at all costs. Just sit next to them. I do that a lot on my house calls, sit where the parents are sitting. I go sit next to them and turn my chair towards them. I avoid sitting directly opposite them because then you might get off on the wrong foot, so to speak.” (CZV 2).*

## Discussion

In this study, CPs were appreciative of the broad perspective offered by the integrated approach for childhood obesity as it takes the individual context and the individual characteristics, possibilities, needs and wishes of children and their parents into account. The frequently used supporting assessment tool was considered to be helpful for gaining insights into psychosocial and lifestyle factors because it offers a tailored approach that can be adjusted to the needs, goals and opportunities of the child and their family. CPs indicated that specific competences could improve their ability to conduct a psychosocial and lifestyle assessment. Specifically, they emphasized that building rapport and a trust-based relationship with children and their parents, along with having the requisite knowledge, skills and attitude were vital for effectively conducting an assessment. This study indicates that CPs require a contextualized and comprehensive understanding of the children with obesity and their circumstances and that the psychosocial and lifestyle assessment is more than merely being a method through which to retrieve information from children and their parents.

The timing when CPs initiate the psychosocial and lifestyle assessment is of critical importance. At that stage, CPs must carefully build rapport and a trusting relationship with the children and their parents. Previous studies have shown that it is absolutely vital that children and their parents feel sufficiently comfortable to engage in these conversations, and that this reduces the risk of drop-out of treatment. The importance of the professional-patient relationship within healthcare is well-established, with research showing that a respectful and trustful relationship between families and healthcare professionals is critical for both fostering open communication and for keeping children and families engaged [32–38]. Or, as Halgunseth et al. observe: “As mutual trust evolves between the family and the program, so will the extent of and commitment to the partnership” [39].

Our study shows that CP’s acknowledge that an open, non-patronizing and non-stigmatizing attitude should be part of initiating and engaging in a constructive conversation about weight, and thereby establishing a meaningful relationship. In accordance with the Dutch national model of integrated care for obesity and in line with previous studies, CPs reported that adopting a non-stigmatizing attitude is a key facilitating factor. Such an attitude encourages children and their families to discuss children’s weight and to raise issues without feeling patronized or judged [13, 40, 41]. This is important because research shows that parents often experience a sense of guilt or failure when discussing their child’s weight, which can lead them to avoid seeking professional support and care [42, 43]. Moreover, this study shows that applying conversational techniques and active listening skills are key elements of adopting an empathic attitude. A previous study confirms that feeling comfortable with such communication skills enables professionals to communicate more sensitively, non-judgmentally and to convey to children and their parents that they should not feel blamed or judged [33]. Moreover, effective professional-patient communication has been linked to patient satisfaction, improved patient adherence and better care outcomes [44]. Furthermore, effective communication by healthcare professionals has the potential to establish rapport and trust [45]. A Dutch profile describes a list of six competences that are required for the role of CP. These competences include being a ‘communicator’. Communication skills include motivational interviewing and solution-focused counselling. This description of the CP role is consistent with our findings, which especially highlighted step two (conduct a broad assessment) of the integrated care approach [46].

Another requirement for conducting a psychosocial and lifestyle assessment was to have sufficient knowledge of the complexity of obesity. This corresponds with a Dutch report about YHC nurses fulfilling the role of CPs [47]. Moreover, a previous study confirms that acknowledging that obesity is a complex disease driven by biological, social, and psychological factors, rather than by personal choices, enhances goal-setting, empathy and the overall level of involvement of the patient [48]. Furthermore, having an affinity with obesity-related issues and extensive experience as a CP were also cited as key factors that both aided the process of conducting a psychosocial and lifestyle assessment and optimized the support and care for children with obesity.

According to this study, the frequently used supporting assessment tool helps CPs to conduct psychosocial and lifestyle assessments, because it stimulates an in-depth conversation about the family’s needs. In addition, it may help to structure the conversation and to delineate the subsequent steps. In order to utilize the supporting assessment tool, an open and non-stigmatizing attitude is required. Implementing this new approach and becoming more confident and comfortable with the necessary knowledge, skills and attitudes is time-consuming [26, 47]. For CPs who fulfil this role in combination with their YHC nurse role without receiving additional time (i.e. several hours per family), this can be challenging. Previous studies have confirmed that having sufficient time is an essential precondition for CPs being able to fulfil their role properly, as time constraints prevent CPs from paying attention to the family’s needs [33, 47]. Moreover, in the national and international childhood obesity guidelines, little attention is paid to the required knowledge, skills and attitudes of the healthcare professionals who are conducting a broad assessment [17, 19, 20]. Emphasizing the complexity of carrying out a psychosocial and lifestyle assessment, as a result of the many elements that can potentially interfere with its successful completion, will lead to better assessments and thereby better support and care for children with obesity and their families. In addition, attention to prevention (for example the obesogenic environment) and the connection between prevention and care remains essential in the integrated care approach [13, 25].

### Strengths and limitations of the study

A strength of this study is that CPs from a variety of professional backgrounds and experience across different municipalities in the Netherlands gave their views on conducting a psychosocial and lifestyle assessment. Moreover, data saturation was achieved, which means that further data collection would not produce different results, but rather would confirm the themes and conclusions. Our study has also some limitations. Firstly, this study focuses on conducting a psychosocial and lifestyle assessment as part of an integrated care approach. This approach is currently being implemented within the Dutch healthcare system, which means that the results of this study may not be generalizable to other healthcare systems in different countries. Furthermore, our results may have been influenced by selection bias, in the sense that it is possible that only those CPs who were positive about their role and more engaged opted to participate. This study initially also planned to interview children and their families. Due to COVID-19 these interviews were cancelled, and, as such, their voices were not heard in the study.

### Practical implications and recommendations for future research

Based on the results of this study a first recommendation is that age-appropriate visual materials should be developed, because these may help to foster productive conversations and a child-friendly atmosphere, and may overcome literacy and language barriers. A second recommendation for practice is to improve the frequently used developed supporting assessment tool. This study pointed to some improvements which can serve as the basis for further developing this tool, including providing an instructional guide for CPs on how to conduct a psychosocial and lifestyle assessment. Moreover, researchers should review extant literature (scientific and grey) and investigate knowledge on conducting a psychosocial and lifestyle assessments. In conjunction with this, it might also be expedient to gain insight into how the supporting assessment tool relates to pre-existing methods of obtaining information from children and their parents in a broader context than childhood obesity, and to take the experiences of professionals into account. In order to successfully manage childhood obesity, the psychosocial and lifestyle assessment and medical assessment should be linked. Thirdly, in order to facilitate CPs’ reporting of the findings, integrating the supporting assessment tool with children’s medical records would provide several benefits: (a) it would provide an unambiguous working method for all professionals, which, in turn, will promote the implementation of this working method, (b) it would help to provide structure when conducting a psychosocial and lifestyle assessment, (c) it would facilitate the immediate reporting of findings digitally rather than using paper notes (d) it would improve the subsequent steps that need to be taken after the psychosocial and lifestyle assessment, as the assessment would ideally be included in the process and carried out over several consultations. Other operating professionals (such as, for example, a paediatrician or YHC professional) within integrated care practice would also benefit, because referral data would be more readily available. Fourthly, in order to enhance CPs’ confidence in conducting psychosocial and lifestyle assessments, it would be beneficial to provide them with continuing education. This could be done through training or peer-to-peer coaching about specific cases, and would focus on the types of knowledge, skills and attitudes that are considered to be important. Fifthly, future research should focus on the conversation that initiates the psychosocial and lifestyle assessment, given that this study showed that a tool can only help to facilitate the conversation, as opposed to replacing or solving it. Finally, the perspectives of the target group, that is, children with obesity and their parents need to be heard, in order to tailor the supporting assessment tool to their needs and wishes.

## Conclusions

This study has demonstrated that the assessment of psychosocial and lifestyle factors, which forms part of the Dutch integrated care approach for childhood obesity, is vital for gaining a broad perspective upon both the individual context and individual characteristics, possibilities, needs and wishes of children with obesity and their parents. In order to effectively conduct this psychosocial and lifestyle assessment sufficient knowledge, confidence in one’s professional skills, adjusting one’s attitude to correspond to each individual child and their parents, as well as conversational techniques is required, which should be supported by continuing education. Further research should focus on improving psychosocial and lifestyle assessments and investigating whether this will lead to improved care and better outcomes, from the perspective of healthcare professionals, children and their parents.

## Supplementary Information

**Additional file 1.**

## Data Availability

All data generated or analysed during this study is included in this published article and Supplementary information files. The data analysed during the current study is available upon reasonable request from the corresponding author (l.koetsier@vu.nl).
