# Supplementary material for: Conducting a psychosocial and lifestyle assessment as part of an integrated care approach for childhood obesity: experiences, needs and wishes of Dutch healthcare professionals
Source: BMC Health Serv Res. 2021 Jun 28;21:611. doi: 10.1186/s12913-021-06635-6 (PMC8238479; doi:10.1186/s12913-021-06635-6)
Supplement: Supplementary file 1 — Additional file 1. [file 12913_2021_6635_MOESM1_ESM.docx]

| **Research question** |  |
| --- | --- |
| 1. Experiences of CPs with regard to conducting a psychosocial and lifestyle assessment as part of an integrated care approach for childhood obesity | **Interview topic guide**   1. CPs’ attitudes towards their role and the vision of the national model 2. Caseload of the CP and perceived differences in the provision of support and care for children with respectively overweight and obesity 3. Conducting a psychosocial and lifestyle assessment; methods used, by whom, how, and where? 4. Themes to discuss with the child and parent when carrying out a psychosocial and lifestyle assessment 5. The (coordinating) process after carrying out a psychosocial and lifestyle assessment 6. Facilitators and barriers related to conducting a psychosocial and lifestyle assessment |
|  | **Identified themes by extracting key words and codes from the coding framework**   1. Initiating a psychosocial and lifestyle assessment 2. Assessment of psychosocial and lifestyle factors 3. CPs’ attitudes towards their role |
| 2. Wishes and needs of CPs with respect to conducting a psychosocial and lifestyle assessment as part of an integrated care approach for childhood obesity | **Interview topic guide**   1. Needs related to the content of psychosocial and lifestyle assessments 2. Needs related to the amount of time and the preferred location for conducting a psychosocial and lifestyle assessment 3. Competences needed to conduct a psychosocial and lifestyle assessment, including the perceived ability needed to conduct a psychosocial and lifestyle assessment |
|  | **Identified themes by extracting key words and codes from the coding framework**   1. Support in initiating and conducting a psychosocial and lifestyle assessment 2. Competences needed by the CP in order to conduct a psychosocial and lifestyle assessment |

**Appendix 1.** Interview topic guides for CPs and the themes that emerged out of the interviews
